# Plant nitrate supply regulates *Erwinia amylovora* virulence gene expression in *Arabidopsis*

**DOI:** 10.1111/mpp.13114

**Published:** 2021-08-12

**Authors:** Mahsa Farjad, Gilles Clément, Alban Launay, Roua Jeridi, Sylvie Jolivet, Sylvie Citerne, Martine Rigault, Marie‐Christine Soulie, Sylvie Dinant, Mathilde Fagard

**Affiliations:** ^1^ Institut Jean‐Pierre Bourgin INRAE AgroParisTech Université Paris‐Saclay Versailles France; ^2^ Laboratoire des Risques Liés Aux Stress Environnementaux Faculté des Sciences de Bizerte, Université de Carthage Bizerte Tunisia; ^3^ Sorbonne Université UPMC Université Paris 06 Paris France

**Keywords:** defence, jasmonic acid, multistress, nitrogen, nonhost, phytopathogenic bacteria, salicylic acid, virulence

## Abstract

We showed previously that nitrogen (N) limitation decreases *Arabidopsis* resistance to *Erwinia amylovora* (Ea). We show that decreased resistance to bacteria in low N is correlated with lower apoplastic reactive oxygen species (ROS) accumulation and lower jasmonic acid (JA) pathway expression. Consistently, pretreatment with methyl jasmonate (Me‐JA) increased the resistance of plants grown under low N. In parallel, we show that in planta titres of a nonvirulent type III secretion system (T3SS)‐deficient Ea mutant were lower than those of wildtype Ea in low N, as expected, but surprisingly not in high N. This lack of difference in high N was consistent with the low expression of the T3SS*‐*encoding *hrp* virulence genes by wildtype Ea in plants grown in high N compared to plants grown in low N. This suggests that expressing its virulence factors in planta could be a major limiting factor for Ea in the nonhost *Arabidopsis*. To test this hypothesis, we preincubated Ea in an inducing medium that triggers expression of *hrp* genes in vitro, prior to inoculation. This preincubation strongly enhanced Ea titres in planta, independently of the plant N status, and was correlated to a significant repression of JA‐dependent genes. Finally, we identify two clusters of metabolites associated with resistance or with susceptibility to Ea. Altogether, our data showed that high susceptibility of *Arabidopsis* to Ea, under low N or following preincubation in *hrp*‐inducing medium, is correlated with high expression of the Ea *hrp* genes in planta and low expression of the JA signalling pathway, and is correlated with the accumulation of specific metabolites.

## Graphical abstract

Under low nitrate, infection of *Arabidopsis thaliana* plants with the phytopathogenic bacterium *Erwinia amylovora* leads to reduced nonhost resistance and increased virulence gene expression.

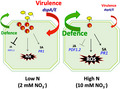

## 1 INTRODUCTION

Plants are usually exposed simultaneously to multiple stresses and many reports have shown that one stress can affect the plant's response to another stress. Indeed, abiotic stresses can interact with biotic stress, either negatively or positively. For example, deficiencies in the soil in mineral elements can trigger an abiotic stress, which in turn can affect plant resistance to pathogens (Dordas, 2008). In particular, a number of reports indicate that nitrogen (N) availability affects plant–pathogen interactions (Fagard et al., 2014).

Nitrogen is an essential nutrient for plant growth and large amounts of N are brought to crops through fertilization worldwide, most often in the form of nitrate. However, N fertilization has strong environmental and economic consequences and affects the severity of many crop diseases. Interestingly, N deficiency can both increase and reduce resistance, depending on the host–pathogen interaction, suggesting that complex mechanisms are at play. For example, addition of N fertilizer to rice reduces resistance to *Magnaporthe oryzae,* a phenomenon described as nitrogen‐induced susceptibility (Ballini et al., 2013). On the other hand, low N supply reduces tomato resistance to *Botrytis cinerea* (Lecompte et al., 2010). Furthermore, N availability can have opposite effects for a given plant species depending on the pathogen. Indeed, low N concentrations in tomato tissues are correlated with a decrease in resistance to *B. cinerea* and an increase in the resistance to *Pseudomonas syringae* and *Oidium lycopersicum* (Hoffland et al., 2000). To further complicate matters, the impact of N for a given pathogen depends on the plant species. For example, in tomato low N decreases the resistance to *B. cinerea* while in strawberry and *Arabidopsis* it increases the resistance to *B. cinerea* (Daugaard et al., 2016; Soulié et al., 2020). Finally, pathogens infecting nonhost plants, such as *P. syringae* pv. *phaseolica* infecting tobacco, are also affected by the form of N supplied to the plant (Gupta et al., 2012). Thus, the use of well‐adapted N fertilization to reduce pathogen attacks is hindered by the multiplicity of effects and by our lack of understanding of the mechanisms involved.

Although the underlying mechanisms remain globally not well understood, previous reports suggest that N availability can affect expression of plant defence, expression of pathogen virulence, and availability of nutrients for the pathogen (Fagard et al., 2014; Zarattini et al., 2021). For example, in *Arabidopsis*, the activities of three enzymes associated with defence, chitinase, chitosanase, and peroxidase, were reduced in low N (Dietrich et al., 2004). In tomato plants infected by *P. syringae*, the phenolic defence compound caffeoyl putrescine was lower in plants grown in low N than in high N (Royer et al., 2013). Concerning the possible effect of N supply on the expression of virulence genes, their expression in vitro is generally higher in minimal growth medium than in rich medium. This is thought to reflect the fact that in planta pathogens are often confronted with a relatively poor environment. Several studies have reported that N availability can affect virulence gene expression in planta and in vitro. In phytopathogenic fungi, several genes encoding putative proteases, avirulence factors or suppressors of plant defence have been shown to be induced by low N conditions in vitro (Snoeijers et al., 2000). In numerous phytopathogenic bacteria, the type III secretion system (T3SS)‐encoding *hrp* bacterial genes are essential virulence factors and have been described in several species to be induced in vitro by low N conditions (Snoeijers et al., 2000). For example, both *Erwinia amylovora* (Ea) and *P. syringae* *hrp* genes showed high expression in low N medium (Wei et al., 1992; Yu et al., 2013). Interestingly, several sources of N repressed Ea *hrp* activation, including amino acids and ammonium.

Ea is a gram‐negative bacterium of the *Enterobacteriaceae* family, causal agent of fire blight, a destructive disease for the Maloideae subfamily of Rosaceae. The main virulence determinants of Ea include the T3SS and an exopolysaccharide (EPS) (Ancona et al., 2013; Holtappels et al., 2016). The T3SS allows phytopathogenic bacteria to inject type III secreted effector proteins (T3Es) inside plant cells to repress defence (Galán & Wolf‐Watz, 2006; He et al., 2004). On the plant side, several defence pathways are involved in defence against Ea, including the salicylic acid (SA)‐ and jasmonic acid (JA)‐dependent pathways and reactive oxygen species (ROS) accumulation (Degrave et al., 2008; Dugé de Bernonville et al., 2012; Launay et al., 2016). Resistance against Ea is considered mostly quantitative, but some pear cultivars, such as Blake's Pride and Potomac, show low susceptibility to Ea (Civetta et al., 2009; Malnoy et al., 2012; Parravicini et al., 2011), and two gene‐for‐gene interactions have been identified in *Malus* (Vogt et al., 2013; Wöhner et al., 2018). Thus, recent studies have focused on the development of elicitors for the protection of apple seedlings and trees against fire blight (Dugé de Bernonville et al., 2012). However, efficiencies of such protection methods in the field are irregular, in particular because they are influenced by environmental conditions.

Recently we showed that low N increases the susceptibility of nonhost *Arabidopsis* plants to Ea (Fagard et al., 2014). In *Arabidopsis*, Ea is able to multiply in a T3SS‐dependent manner and triggers similar defence pathways as in apple seedlings (Degrave et al., 2008, 2013). To understand the interactions between plant physiology and bacterial infection in a context of N limitation, we studied plant defence activation, metabolite accumulation, and bacterial virulence gene expression in *Arabidopsis* plants grown in low or high N and infected with Ea. Genetic and physiological dissection were performed to determine whether modifications of known virulence factors, defence signalling pathways, or metabolite accumulation are responsible for the higher susceptibility in low N.

## 2 RESULTS

### 2.1 Low N favours in planta bacterial titres independently of bacterial nutrition

**FIGURE 1:**
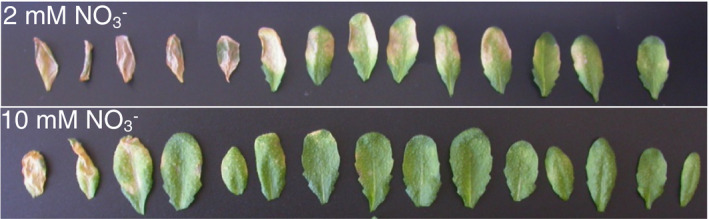
Plants grown under high nitrate are less susceptible to *Erwinia amylovora* (Ea). *Arabidopsis* plants grown under low or high ${\text{NO}}_{3}^{‐}$ were inoculated with wildtype Ea. Leaves of five plants are shown 7 days postinoculation. Mock‐inoculated control leaves showed no symptom (not shown). The leaves are ordered according to the severity of visible symptoms. The experiment was conducted twice with similar results. Ea bacterial titres 24 hr postinoculation were published previously (Fagard et al., 2014)

**FIGURE 2:**
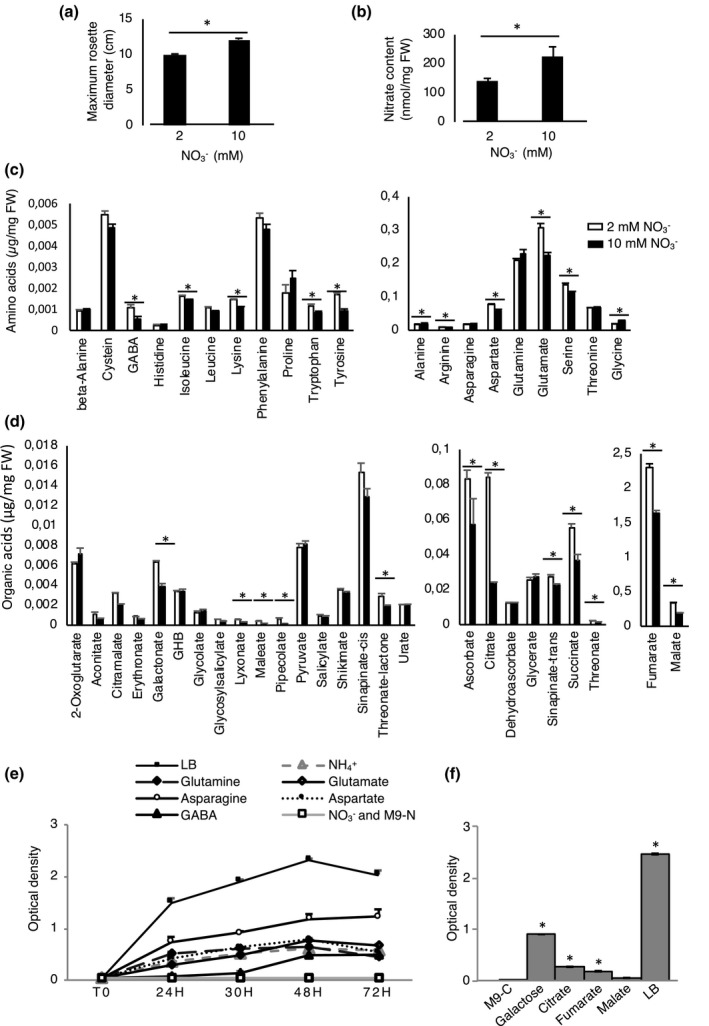
Comparison of physiological traits in 5‐week‐old plants grown under low or high nitrate conditions. (a) Maximum rosette diameter plants (cm). (b) Leaf nitrate content (nmol/mg fresh weight [FW]). (c, d) Comparison of the raw values of amino acids and organic acids in the rosette leaves. Mean and *SE* of four individual plants are presented. (a–d) Asterisks indicate significant differences according to analysis of variance (ANOVA), *p* < 0.05. (e) Dynamics of Ea growth in vitro. The graph shows the optical density (OD_600_) of Ea incubated in a minimal medium containing 5 mM of various N sources at different time points. The precise medium content is described in Table S1b. N sources: asparagine, aspartate, glutamine, glutamate, γ‐aminobutyric acid (GABA), ${\text{NO}}_{3}^{‐}$ or ${\text{NH}}_{4}^{+}$. M9−N, negative control with no N source; LB, Luria‐Bertani (rich) medium. Similar results were obtained for two independent experiments. One representative experiment is shown and for each independent experiment mean and *SE* are presented (*n* = 3). (f) Capacity of Ea to use selected organic acids as carbon source. The graph shows the OD_600_ at 72 hr of culture with the indicated carbon (C) source. C sources: galactose, citrate, fumarate, malate. M9−C, negative control with no carbon source; LB, rich medium. Asterisks indicate significant differences from M9−C condition (Mann–Whitney test ; *p* < 0.05)

We showed previously that wildtype *Arabidopsis thaliana* Col‐0 plants grown in low N (2 mM ${\text{NO}}_{3}^{‐}$) are more susceptible to Ea than plants grown in high N (10 mM ${\text{NO}}_{3}^{‐}$) (Fagard et al., 2014). Indeed, in planta bacterial titres and symptom intensity were both lower in high N (Figure 1). These conditions were previously described as mildly limiting (2 mM ${\text{NO}}_{3}^{‐}$) and nonlimiting (10 mM ${\text{NO}}_{3}^{‐}$) growth conditions (Lemaitre et al., 2008). Consistently, for plants grown in low N we found a moderate but significant reduction of rosette diameter (Figure 2a) and a moderate but not significant reduction in biomass (Figure S1). To understand how N plant nutrition affects bacterial in planta titres, we first tested the hypothesis that nitrate availability affects nutrient availability for bacteria inside the leaves. As expected, ${\text{NO}}_{3}^{‐}$ content in leaves was significantly more elevated in plants grown in high N (Figure 2b). Total amino acid content measurement did not show a statistical difference between plants grown in high and low N (not shown). A gas chromatography‐mass spectrometry (GC‐MS) analysis of total metabolites indicated that there were slight and significant differences in the balance of several amino acids between plants grown in high and low N (Figure 2c). Furthermore, several organic acids, namely citrate, fumarate, succinate and malate, accumulated more in low N, especially citrate, which showed a 72% reduction in accumulation in plants grown in high N (Figure 2d), However, most of these modifications were moderate, probably reflecting a modification of the metabolic fluxes in these conditions (Krapp et al., 2011). Altogether, our results for the Col‐0 accession are consistent with previous results obtained for the Wassilewskija accession (Fagard et al., 2014; Lemaitre et al., 2008).

Thus, the main impact of low N on the physiology of Col‐0 plants in our conditions is a reduced rosette diameter, a decrease in total leaf ${\text{NO}}_{3}^{‐}$, a slight modification in the balance of amino acids, and an increase in organic acids. Altogether, the physiology of 5‐week‐old Col‐0 rosettes was only moderately impacted when plants were grown in low N.

Previous reports indicate that Ea cannot reduce nitrate to nitrite and thus cannot use nitrate as a source of N, unlike most enterobacteria (Paulin, 2000). Thus, we cannot explain the differences in bacterial populations in plants grown in low and high N by the fact that bacteria have less ${\text{NO}}_{3}^{‐}$ available in low N conditions because Ea cannot assimilate ${\text{NO}}_{3}^{‐}$. On the other hand, plants grown in low N showed a different balance in relative amino acid and organic acid abundance (Figure 2c), and it was suggested previously that aspartate is an important source of N for Ea in host plants (Paulin, 2000). To determine whether Ea uses amino acids with different efficiencies in vitro, we compared Ea growth in rich (Luria‐Bertani, LB) versus minimal medium (M9) containing various N sources (Figure 2e). We tested nitrate, ammonium, and some of the most abundant amino acids found in *Arabidopsis* leaves, that is, asparagine, aspartate, glutamine, glutamate, and γ‐aminobutyric acid (GABA). As expected, Ea was not able to use nitrate as an N source for in vitro growth but was able to use ammonium and all the amino acids tested. Contrary to what was suggested previously (Paulin, 2000), we found that asparagine was a more favourable N source for Ea growth than the other N sources tested. However, there were no differences in asparagine levels in plants grown in low and high N. Concerning organic acids, we found that Ea can use fumarate and citrate as carbon sources, but that they are poor carbon sources compared to galactose (Figure 2f). Furthermore, malate, one of the major organic acids in *Arabidopsis thaliana* leaves (Figure 2d), cannot be used as a carbon source by Ea (Figure 2f). Thus, the amino acids and organic acids that show an increase in leaves of plants grown in low N do not correspond to the preferred N and C sources, respectively, used by Ea in vitro. However, because Ea develops in the intercellular spaces, it is possible that nutrient levels observed in whole leaves do not reflect resources actually available for bacteria. Furthermore, because infection can affect plant metabolite content, we also analysed metabolite content following infection (see below).

### 2.2 ROS accumulation is affected by N availability

**FIGURE 3:**
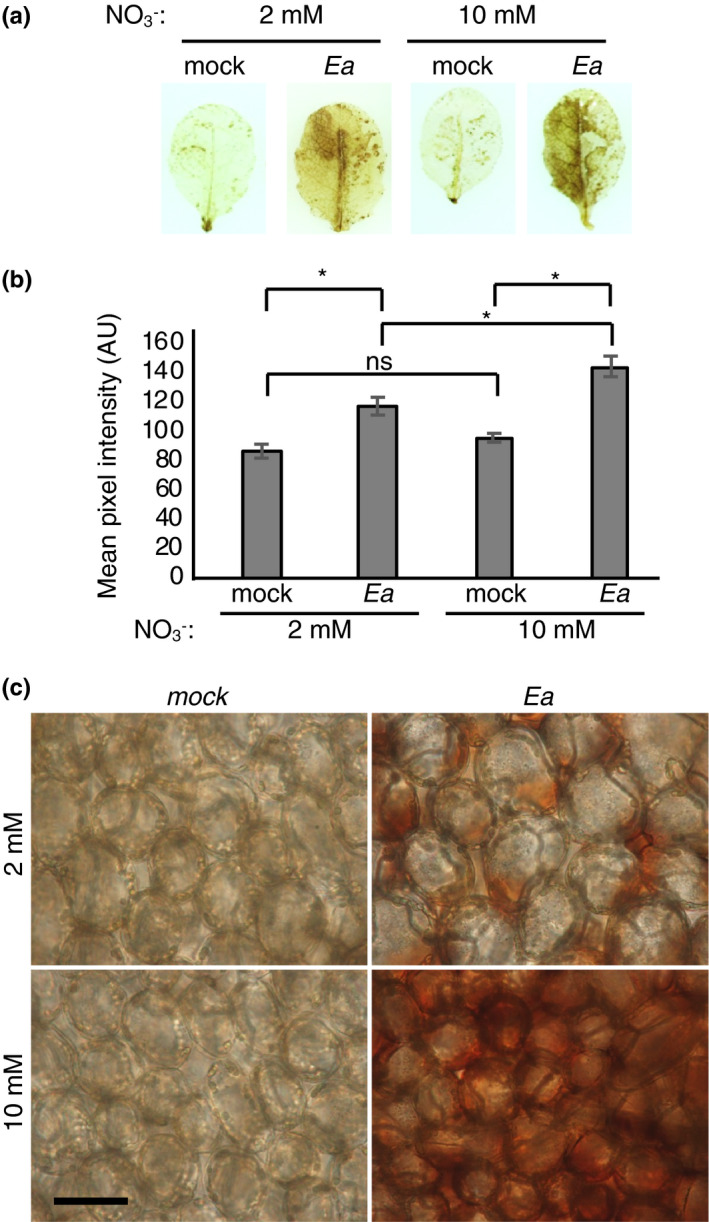
*Erwinia amylovora* (Ea) induces apoplastic accumulation of H_2_O_2_ in plants grown in high N. (a–c) Leaves of 5‐week‐old plants were infiltrated with water (mock) or wildtype Ea. The right half of each leaf was left uninoculated as a control. Diaminobenzidine (DAB) staining was performed to reveal H_2_O_2_ accumulation. (b) Mean intensity of DAB staining of the inoculated half of 10 leaves per condition. Asterisks indicate significant differences between conditions (Mann–Whitney test, *p* < 0.05). (c) Localization of H_2_O_2_ deposits in DAB‐stained leaves was observed using a Nikon Microphot‐FXA microscope. Bar size, 50 μm

ROS accumulation is a common defence response to avirulent pathogens and to nonadapted pathogens such as Ea (Launay et al., 2016). To determine whether N supply had an effect on ROS accumulation following infection, we used diaminobenzidine (DAB), which forms a brown precipitate in the presence of H_2_O_2_. As expected, the mock‐inoculated control leaves displayed only weak brown staining that was not significantly different between the two N conditions (Figure 3a,b). In contrast, we observed strong brown staining in Ea‐inoculated leaves of plants grown in both high and low N conditions (Figure 3a). Interestingly, the intensity of DAB staining was slightly lower in plants grown in low N, which are more susceptible to Ea (Figure 3b,c). Altogether, our results indicate that higher susceptibility to Ea of plants grown in low N is correlated with slightly lower ROS accumulation.

### 2.3 JA‐dependent defence plays a key role in N modulation of susceptibility

To further study the impact of N supply on *Arabidopsis* defence signalling pathways in response to Ea infection, we monitored the expression of several genes dependent on the SA and JA signalling pathways shown previously to be induced in response to infection by Ea (Moreau et al., 2012).

**FIGURE 4:**
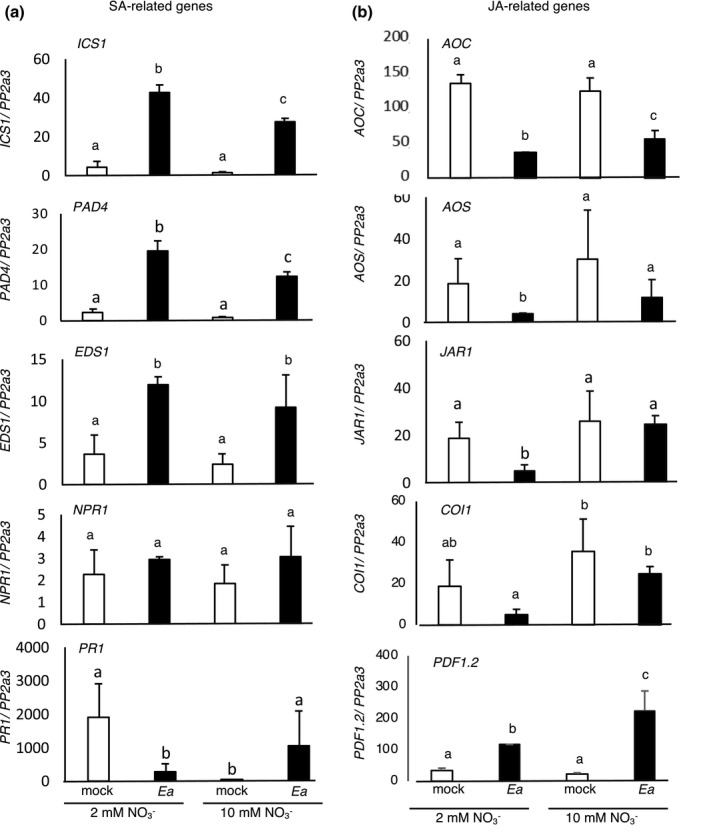
Plant defence gene expression analysis. Salicylic acid (SA)‐ (a) and jasmonic acid (JA)‐related (b) gene expression in 5‐week‐old wildtype plants grown under low or high ${\text{NO}}_{3}^{‐}$. Plants were inoculated with water (mock) or *Erwinia amylovora* (Ea) and harvested 6 hr postinoculation (hpi) for RNA extraction and quantitative reverse transcription PCR analysis. The values indicate gene expression in arbitrary units calculated relative to the constitutive *PP2a3* gene expression level. Each bar corresponds to the average gene expression in three biological replicates. Different letters indicate a significant difference between two conditions (Mann–Whitney test; *p* < 0.05). Experiments were repeated three times with similar results

For the SA pathway, we studied the expression of *ICS1*, involved in SA biosynthesis, *PAD4*, *NPR1*, and *EDS1*, regulators of SA‐dependent signalling, and finally *PR1*, a downstream marker of SA signalling. We monitored the expression of these genes by quantitative reverse transcription PCR (RT‐qPCR) in 5‐week‐old *Arabidopsis* plants grown in low or high N, mock‐ or Ea‐inoculated and sampled at 6 hr postinoculation (hpi) (Figure 4a). For all genes except *PR1*, the level of expression in control leaves was similar in the two nutritional regimes. In response to Ea inoculation, *NPR1* showed no induction while *ICS1*, *PAD4*, and *EDS1* were induced in plants grown both in low and high N (Figure 4a). For *ICS1* and *PAD4*, the level of expression following infection was slightly higher in plants grown in low N. Concerning *PR1*, we found an induction following infection only in leaves of plants grown in high N (Figure 4a). On the contrary, the expression of *PR1* following Ea inoculation was repressed in plants grown in low N, as observed transiently in apple flowers by Pester et al. (2012). At a later timepoint (24 hpi), we observed an induction of *PR1* expression in plants grown in low N, in response to Ea; however, the expression level remained lower than in plants grown in high N (not shown).

**FIGURE 5:**
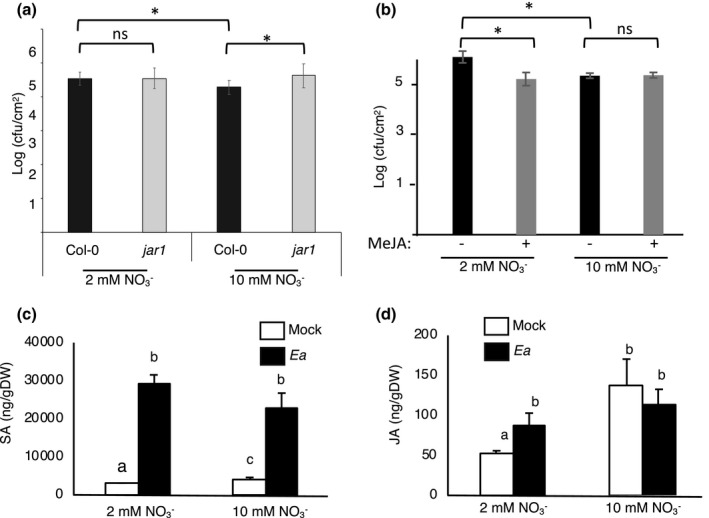
Role of jasmonic acid (JA) in the impact of N on susceptibility. (a, b) Plants were grown under low or high nitrate conditions for 5 weeks, then inoculated with wildtype *Erwinia amylovora* (Ea) and harvested 24 hr postinoculation (hpi). (a) Wildtype Ea bacterial titres at 24 hpi in the JA‐deficient *jar1‐1* mutant compared to wildtype Col‐0 plants. (b) Leaves were sprayed with 0.5 mM methyl jasmonate (MeJA, +) or dimethyl sulphoxide (DMSO, −) 48 hr prior to being inoculated by Ea. (a, b) Asterisks indicate significant differences between indicated conditions according to Mann–Whitney tests, *p* < 0.05. Quantification of salicylic acid (SA; c) and JA (d). Leaves of 5‐week‐old plants grown in low or high N were infiltrated with water (white bar) or Ea wildtype strain (black bar) and harvested 24 hpi. Columns with different letters are statistically different according to Mann–Whitney tests, *p* < 0.05. Bars correspond to *SE*

For the JA signalling pathway we analysed *AOS* and *AOC*, encoding JA biosynthesis enzymes, *JAR1*, required for conjugation of JA to Ile to form its bioactive form, *COI1*, required for JA perception, and *PDF1.2*, a downstream marker of the JA pathway. The level of expression of these genes in mock‐inoculated leaves of plants grown in low or high N was similar (Figure 4b). Following Ea inoculation *AOS*, *JAR1*, and *COI1* were repressed only in plants grown in low N while *AOC* and *PDF1.2* were respectively repressed and induced both in low and high N. All five genes showed lower expression levels in plants grown under low N in infected plants (Figure 4b). To confirm the putative role of JA signalling on the susceptibility of *Arabidopsis* to Ea, we analysed Ea titres in the corresponding *Arabidopsis* mutant. We found that the *jar1* mutant, affected in JA signalling, was more susceptible to Ea in high N, although the differences were low (Figure 5a). On the contrary, the SA‐deficient *nahG* transgenic line was not more susceptible to Ea under high N conditions (Figure S2). Furthermore, we found that pretreatment of *Arabidopsis* leaves with methyl jasmonate (Me‐JA) led to a decrease of Ea titres 24 hpi in plants grown in low N but not in high N (Figure 5b). These results confirm the importance of the JA pathway in the modulation of susceptibility by ${\text{NO}}_{3}^{‐}$ availability for plants.

To corroborate these results, we measured the level of hormone accumulation in leaves of plants grown in low or high N following mock‐ or Ea‐inoculation. SA and JA levels were measured in leaves by high performance liquid chromatography (HPLC) as described previously (Zarattini et al., 2017). In control plants grown in high N both SA and JA accumulated slightly more than in control plants grown in low N (Figure 5c,d). Following inoculation with Ea, SA accumulated significantly and to comparable levels in plants grown in low and high N. Following inoculation with Ea, an increase in JA levels was observed only in plants grown in low N, but JA levels were not significantly different between plants grown in low and high N. These data indicate that plant N status affects mainly basal levels of SA and JA hormones. Furthermore, we found no direct correlation between hormone levels in low and high N and gene expression levels.

### 2.4 N availability to the plant affects bacterial virulence gene expression in planta

**FIGURE 6:**
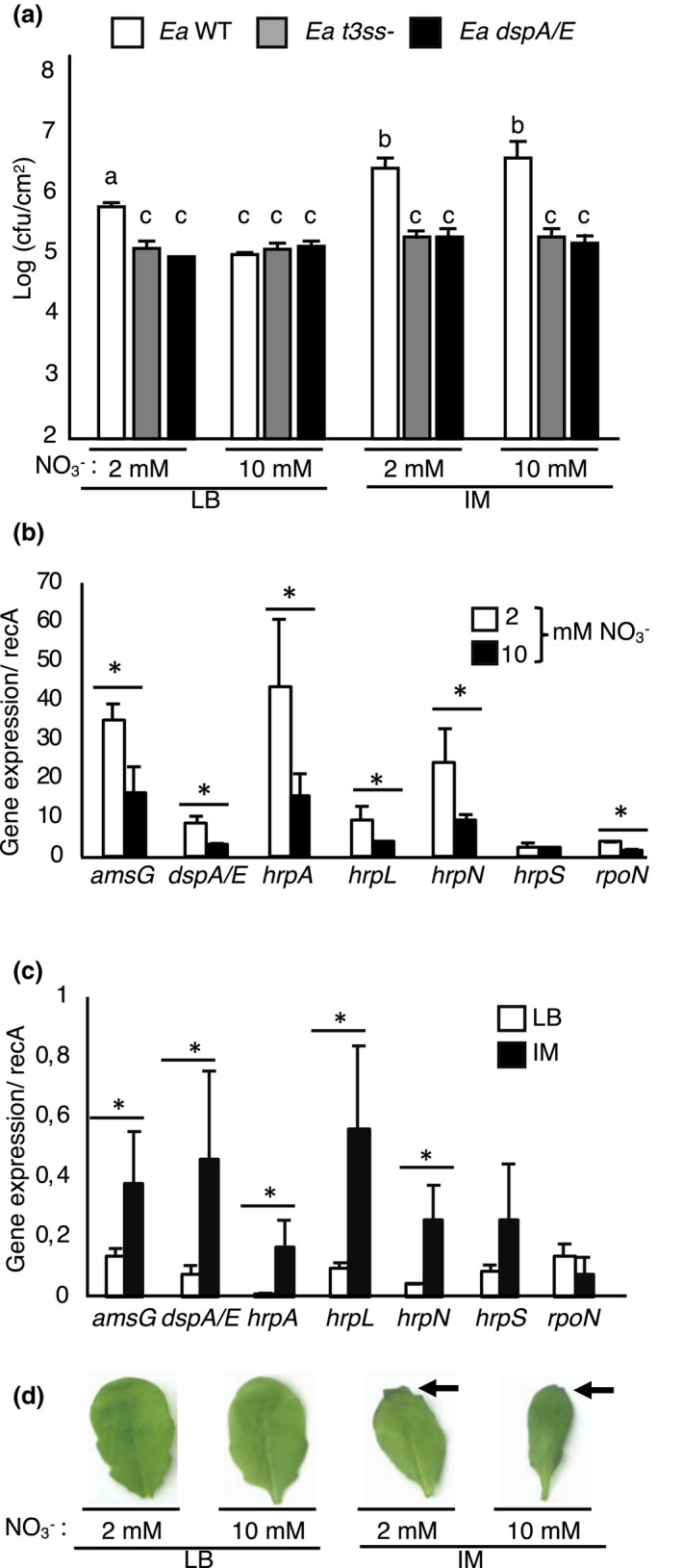
High N availability attenuates type III secretion system (T3SS) expression. (a) Bacterial titres of wildtype (WT) *Erwinia amylovora* (Ea) and mutant strains in *Arabidopsis* leaves grown in low or high N at 24 hr postinoculation (hpi). Bacteria were incubated for 6 hr in Luria‐Bertani medium (LB) or inducing medium (IM) prior to syringe‐inoculation of bacteria. The titres of Ea WT (white), *t3ss* (grey), and *dspA/E* (black) mutant strains in *Arabidopsis* leaves were measured at 24 hpi. Values correspond to the mean value of three biological replicates. (b) In planta expression of Ea genes at 24 hpi in plants grown in low or high N, following preincubation of Ea in LB. (c) In vitro expression of Ea genes following incubation in LB or IM. Expression is normalized to *recA* and represents mean values of two replicates of bacterial suspension. (a–c) Asterisks and different letters indicate significant differences according to the Mann–Whitney test, *p* < 0.05. Similar results were obtained for two independent experiments. (d) Ea‐inoculated leaves of plants grown in low or high N. Ea was preincubated in IM or in LB. Pictures were taken 24 hpi. Arrows indicate leaves with apparent wilting at the margins

Ea growth in host leaf tissue is correlated with strong and early expression of its T3SS‐encoding *hrp* genes and particularly of DspA/E, the major Ea T3E (Pester et al., 2012). In vitro, the nature and the quantity of N available for Ea are known to affect expression of its *hrp* genes (Wei et al., 1992). We therefore wondered whether N availability for plants affected the capacity of Ea to express its T3SS during the infection process. We first determined the requirement for the T3SS under different N regimes by analysing bacterial titres of a T3SS‐deficient Ea mutant and a DspA/E‐deficient Ea mutant in planta in comparison with the wildtype Ea strain. For these experiments, Ea was precultured in rich medium (Luria‐Bertani, LB) before inoculation (Figure 6a; LB panel) and, as expected, for the wildtype Ea strain in planta bacterial titres were lower in plants grown in high N (Fagard et al., 2014). The T3SS‐ and DspA/E‐deficient strains showed lower titres than wildtype Ea in low N as expected but surprisingly not in high N. One possible explanation for the similar titres observed in high N for the wild type and the T3SS‐deficient Ea strains could be that the T3SS is poorly expressed in plants grown in high N conditions.

We then monitored the effect of N on the expression of Ea virulence genes in *Arabidopsis* plants grown in low and high N. Our results indicated that *hrpN*, *hrpL*, *rpoN*, *dspA/E*, and *amsG* were significantly more expressed in plants grown in low N than in high N (Figure 6b). We detected no significant effect of the N regime on the in planta expression of *hrpS*. These results indicate that the low availability of N for plants increases the expression of several genes involved in Ea pathogenicity.

To determine whether the expression of virulence genes was limiting for Ea in *Arabidopsis*, we preincubated the bacteria before inoculation in minimal medium containing galactose, hereafter referred to as inducing medium (IM), instead of the usual preincubation in rich medium (LB). We analysed the expression of *rpoN*, *hrpL*, and *hrpS*, encoding sigma factors that control *hrp* gene expression; *hrpA*, which encodes a structural component of the T3SS; *dspA/E*, encoding a T3E; *hrpN*, which encodes a harpin; and *amsG*, which is involved in exopolysaccharide biosynthesis (Ancona et al., 2013). When bacteria were grown in vitro in IM, the majority of these genes were significantly more expressed compared to the control condition (LB) (Figure 6c). Only *hrpS* and *rpoN* were not affected by the incubation medium. In planta bacterial numbers were then quantified 24 hpi (Figure 6a, IM panel). The preincubation of wildtype Ea in IM led to a significant increase in the bacterial titres in comparison to bacteria preincubated in LB, which was independent of N supply (Figure 6c). Furthermore, *Arabidopsis* leaves were more susceptible to wildtype Ea preincubated in IM than to Ea preincubated in LB, as shown by the appearance of early symptoms at 24 hpi (Figure 6d). By contrast, in planta titres of the T3SS‐ and DspA/E‐deficient mutants were not affected by preincubation in IM (Figure 6c). These results indicate that higher Ea titres following preincubation in IM before inoculation are T3SS‐ and DspA/E‐dependent.

Altogether, our results indicate a correlation between expression levels of Ea virulence genes, in planta bacterial titres, and *Arabidopsis* susceptibility to Ea and show that the increase in bacterial titres requires the presence of the T3SS and DspA/E.

### 2.5 Preincubation of Ea in IM impacts defence expression

**FIGURE 7:**
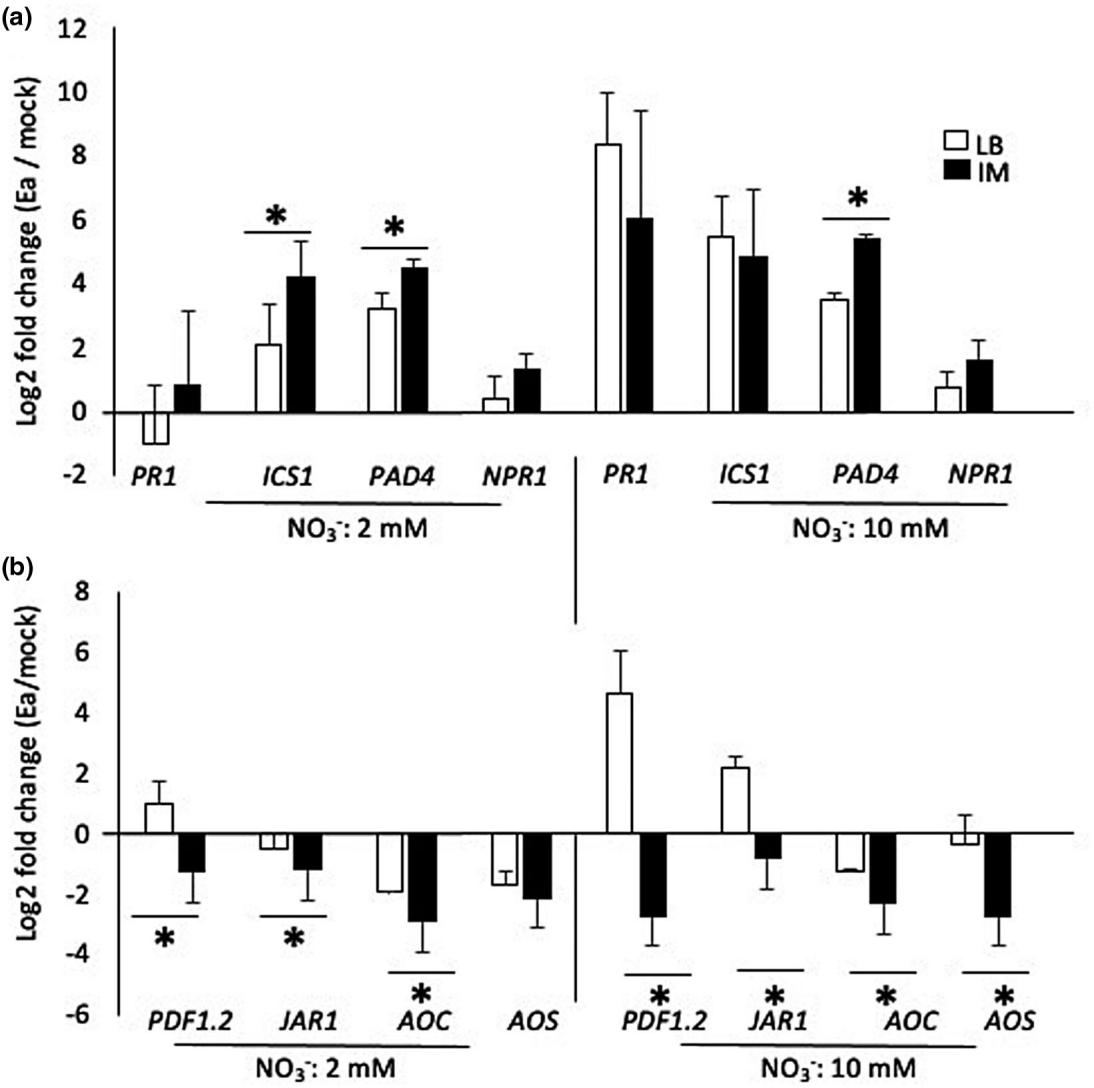
Impact of *Erwinia amylovora* (Ea) *hrp* gene preinduction on *Arabidopsis* defence gene expression. Fold induction of salicylic acid (SA)‐ (a) and jasmonic acid (JA)‐ (b) related genes in Ea‐inoculated leaves versus mock‐treated leaves. *Arabidopsis* plants were grown in low or high N, infiltrated with water (mock) or wildtype Ea. Bacteria were preincubated in Luria‐Bertani medium (LB) or induction medium (IM) prior to infection. Leaves were sampled 6 hr postinoculation (hpi) for RNA extraction and quantitative reverse transcription PCR analysis. Asterisks indicate a significant difference between indicated conditions according to Mann–Whitney tests, *p* < 0.05

We hypothesized that IM preincubation, which led to a higher expression of the T3SS and higher bacterial titres, led to the repression of defence. Five‐week‐old *Arabidopsis* plants grown in low or high N were mock‐inoculated or inoculated with Ea preincubated in LB or IM. We monitored several SA‐ and JA‐marker genes expression 6 hpi (Figure 7a,b). The level of induction of SA pathway marker genes was only mildly affected by the preincubation of Ea in IM (Figure 7a). Only *ICS1* and *PAD4* showed a slight increase in expression in response to bacteria preincubated in IM. On the contrary, expression of JA pathway genes was strongly affected by IM preincubation (Figure 7b). Indeed, the JA biosynthesis genes *AOS* and *AOC* were more strongly repressed in response to bacteria preincubated in IM than in response to bacteria pretreated with LB. Furthermore, *JAR1* and *PDF1.2* were both repressed in response to bacteria preincubated with IM whereas they were either induced or not modulated in response to bacteria pretreated in LB (Figure 7b). These results indicate that the main effect of preincubation of Ea in IM is on the JA signalling pathway, suggesting that JA pathway signalling is one of the main targets of Ea virulence genes.

Altogether, our results show that preincubation of Ea in IM medium leads to high expression of Ea *hrp* genes in planta, and to a repression of JA‐dependent defences.

### 2.6 Preincubation of Ea in IM impacts metabolite accumulation

**FIGURE 8:**
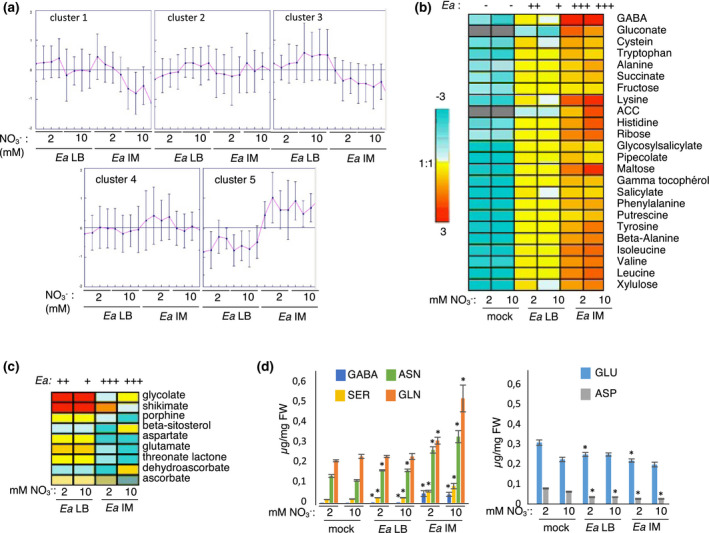
Identification of metabolites correlated with different *Erwinia amylovora* (Ea) titres. Plants were grown under low or high nitrate conditions for 5 weeks, inoculated by Ea (preincubated in Luria‐Bertani medium (Ea LB) or induction medium (Ea IM) or mock‐treated (mock). Leaves were harvested 24 hr postinoculation (hpi) and metabolite analysis was performed by gas chromatography‐mass spectrometry (GC‐MS). (a) Cluster analysis of metabolomics data. All the metabolites detected by GC‐MS were divided into five major clusters by GENESIS software. Heat map of relative metabolite levels in a cluster correlated with susceptibility (b) or resistance (c) to Ea based on the individual metabolite proportions. The level of susceptibility to Ea is indicated using a qualitative scale based on Ea titres shown in Figure 6a. The colour of each cell corresponds to the log_2_ ratio of each metabolite relative to median centre for a given metabolite. Ratio = 1, yellow; ratio < 1, blue; ratio > 1, red. Hierarchical clustering was performed using GENESIS software. (d) Leaf content of major amino acids 24 hr following mock‐ or Ea inoculation. The left panel corresponds to selected amino acids that accumulate more following inoculation, the right panel to selected amino acids that accumulate less following infection. Asterisks indicate significant differences with the corresponding mock condition (Mann–Whitney test, *p* < 0.01)

Pathogen infection leads to important modifications in the metabolome and although less data is available than for the transcriptomic response to biotic stress, some molecules are known to accumulate during resistance and others during susceptibility (Castro‐Moretti et al., 2020; Fagard et al., 2014). To determine whether the high in planta Ea titres observed following IM pretreatment were associated with a modification in levels of specific metabolites, a metabolomic analysis was carried out by GC‐MS (Figure 8). For this purpose, 5‐week‐old *Arabidopsis* plants grown in low or high N were inoculated with Ea, preincubated in LB or IM, and sampled 24 hpi. Our results show an elevated modification in several plant metabolites, including sugars, amino acids, organic acids, and other compounds related to defence (Figure 8 and Figure S3). We performed hierarchical clustering analysis to group metabolites that accumulated under each treatment and found five major clusters (Figure 8a). To identify metabolites susceptible to having a role in the increase in susceptibility to Ea observed when bacteria were preincubated in IM, we studied cluster 5, which corresponds to 24 metabolites that accumulated more when plants were more susceptible (i.e., Ea preincubation in IM). In this cluster, we found many amino acids (alanine, cysteine, GABA, β‐alanine, histidine, leucine, isoleucine, valine, lysine, tyrosine, tryptophan, and phenylalanine) and several sugars (fructose, maltose, ribose, and xylulose) (Figure 8b). Thus, preincubation of bacteria in inducing medium led to specific modifications in metabolite accumulation, in particular with strong GABA accumulation. In contrast, we found that cluster 3 contained metabolites that accumulated more when plants were more resistant to Ea (Figure 8c). In this cluster, we found several organic acids, such as shikimate and dehydroascorbate, several amino acids, and β‐sitosterol, known to be involved in plant immunity (Aboobucker & Suza, 2019; Wang et al., 2012). Interestingly we found amino acids in both cluster 3 and cluster 5. Indeed, among the most abundant amino acids present in *Arabidopsis* leaves, several were found to increase following Ea inoculation (Figure 8d, left panel) and a few were found to decrease following Ea inoculation (Figure 8d, right panel).

Altogether we identified several metabolites that were correlated with resistance or susceptibility, but the biological significance of this accumulation remains to be determined.

## 3 DISCUSSION

We showed previously that the susceptibility of nonhost *Arabidopsis* to the necrotroph Ea is reduced in high N, with lower titres of Ea than in low N (Fagard et al., 2014). Interestingly, in *Malus* genotypes application of N fertilizers is on the contrary known to increase susceptibility to Ea. Although the underlying mechanisms are not known, it is thought that increased susceptibility to fire blight under high N fertilizer conditions in *Malus* is due to the increased presence of growing tissue (Fallahi & Mohan, 2000).

Our first hypothesis to explain the higher titres of Ea in nonhost *Arabidopsis* plants grown in low N was that defence activation was weaker in these conditions. In the present study, we show that the higher Ea titres found in low N were associated with lower JA signalling. SA signalling was also slightly affected by N availability but the differences could not explain the differences in bacterial titres. Most genes associated with the JA pathway were repressed by Ea, with the exception of the downstream JA‐responsive gene *PDF1.2*, which was induced by Ea. However, this induction was very weak in low N, conditions where Ea titres were high. This repression of the JA signalling pathway suggests that it could be the main target of Ea to suppress plant defence. This hypothesis is supported by the observation that in susceptible *Malus* genotypes, a strong downregulation of the JA pathway has been reported (Dugé de Bernonville et al., 2012). Furthermore, in *Malus* this downregulation of the JA pathway by Ea was not observed with an Ea T33S‐deficient mutant, indicating that repression of the JA‐pathway is T3SS‐dependent. The authors proposed that repression of the JA pathway is a critical step in the infection process of *Malus* by Ea. In the present study, we also observed that higher Ea titres, observed in low N, were associated with low ROS accumulation, suggesting a role of ROS formation in resistance of *Arabidopsis* grown in high N, although further experiments would be required to demonstrate this link. This is consistent with our recent data showing that higher susceptibility of the *nrt2.6* mutant to Ea was correlated with lower ROS accumulation (Dechorgnat et al., 2012). Thus, altogether, our data suggest that the higher bacterial titres observed in low N are linked to a repression of both the JA pathway and ROS. However, whether or not there exists a link between ROS accumulation and the JA pathway remains to be established. Consistent with our observations, we found that a *jar1* mutant, deficient for JA signalling, was more susceptible to Ea in high N and that pretreatment with Me‐JA led to increased resistance of plants grown under low N. Thus, our data show that in high N, JA‐dependent plant defence limits Ea titres in planta.

The second hypothesis tested was that N supply to plants affects pathogen nutrition in planta. Indeed, pathogens need to find a source of N once inside the plant tissue and modifications of N supply to the plant could affect N sources available for exploitation as a nutritional source by the pathogen. Our metabolomics analysis of *Arabidopsis* leaves showed that although we did find significant differences in the metabolite content of noninfected leaves, concerning amino acids, sugars and organic acids, these differences remained moderate. Furthermore, the amino acids that showed a higher accumulation in leaves of plants grown under low N did not correspond to the preferred N sources of Ea in vitro. Concerning organic acids, which can be used as a carbon source by the bacteria, only citrate showed a strong increase in low N conditions, suggesting that high citrate could explain high bacterial titres in low N. However, the high bacterial titres obtained after IM preinduction of virulence genes are not associated with high citrate. Although further investigation is required to answer this question, these results suggest that other mechanisms are at play. Furthermore, our data concerns only whole leaf extracts and it would be interesting to measure apoplastic metabolite contents because Ea is an extracellular pathogen. It has been previously proposed that aspartate could be a potential source of N for Ea in planta because it was the major amino acid (58%) in apple shoots (Paulin, 2000). In *Arabidopsis* leaves, aspartate was also one of the major amino acids in noninfected plants. However, leaves grown in low or high N showed very similar aspartate contents and, in vitro, aspartate was not the best source of N for Ea growth. Thus, although we cannot exclude an impact of N on nutrient availability for Ea growth in planta, this is unlikely to be the sole explanation for the differences in Ea titres observed in plants grown in low and high N.

Following plant infection with Ea, we found significant modifications in leaf amino acid content, indicating a specific metabolite reprogramming during plant infection. The levels of major amino acids, such as glutamate and aspartate, decreased in response to infection while other amino acids increased, including aromatic and branched chain amino acids, which is in accordance with a previous study that showed that several aromatic amino acids, tyrosine, tryptophan, and phenylalanine, accumulated significantly following *Arabidopsis* infection by *P. syringae* (Ward et al., 2010). This was particularly true for the levels of GABA and lysine, which increased strongly following Ea infection. Previous studies have reported that GABA accumulates in plants in response to a variety of abiotic and biotic stress as well as pathogen attack (Seifi et al., 2013). For example, GABA is synthesized by *Arabidopsis* in response to infection by *P. s* *yringae* (McCraw et al., 2016). In contrast, we observed that aspartate and glutamate levels decreased in response to Ea infection. Similarly, Ward et al. (2010) have shown that the levels of aspartate were reduced at 18 hpi in plants infected by *P. syringae* pv. *tomato* compared to mock‐inoculated plants. The depletion in glutamate and aspartate could be linked to their role as precursors of GABA (Hildebrandt et al., 2015) and lysine (Azevedo et al., 2006), respectively. Conversely, the reduction in aspartate and glutamate levels could reflect their use by Ea as a nutritional source. It is also possible that the plant cells and bacteria compete for the use of aspartate and glutamate, for plant defence, and for bacterial nutrition and defence avoidance, respectively. Further investigation is required to distinguish between these hypotheses. However, although metabolic fluxes may play an important role during the interaction, the differences in amino acid levels we observed do not seem to explain the differences in Ea titres in low and high N.

The third hypothesis we tested was that Ea virulence gene expression in planta was affected by N supply to plants. This hypothesis was supported by previous data indicating that in vitro low N conditions induce expression of the T3SS‐encoding *hrp* gene cluster (Gaudriault et al., 1997; Wei et al., 1992) and by the observation that a T3SS‐deficient Ea mutant showed similar titres in high N as the wildtype Ea strain instead of reduced titres as expected. We observed that several Ea virulence genes were significantly more expressed in planta in low N than in high N. This included the *hrpL* gene, known as the master regulator of the *hrp* gene cluster (Bush & Dixon, 2012). Interestingly, *rpoN*, which was previously found to play a role in N assimilation in addition to *hrp* gene regulation, through the regulation of *hrpL* (Bush & Dixon, 2012), was also more expressed in plants grown in low N. It was reported that *rpoN* is required for Ea virulence in pear (Ancona et al., 2013). These results suggest that *rpoN* could play a key role in planta by regulating T3SS expression through the perception of reduced N availability. However, the current state of the art has not allowed precisely which factors allow in planta activation of *hrp* genes to be determined.

The last part of our study consisted of the preinduction of Ea virulence genes prior to infection of *Arabidopsis* leaves. This pretreatment led to higher Ea titres in planta independent of the N regime, indicating that *hrp* gene expression was likely to be limiting for Ea in *Arabidopsis* leaves. Furthermore, higher Ea titres were associated with a strong repression of all the genes of the JA pathway that we tested, and a moderate increase in the expression of several genes of the SA pathway. This is consistent with a major role for the JA pathway in the interaction of Ea with host plants. Furthermore, although the SA pathway is strongly induced by Ea infection it probably does not have a strong role in plant defence against Ea, as suggested by our previous results using the SA‐deficient *sid2 Arabidopsis* mutant (Degrave et al., 2008). Our results also strongly suggest that the T3SS of Ea mainly targets the JA signalling pathway, as previously suggested by other studies (Dugé de Bernonville et al., 2012). However, we cannot exclude the hypothesis that the repression of the JA pathway is the result of an increase in SA signalling in *Arabidopsis* plants that are more susceptible to Ea.

Our metabolomics analysis allowed us to identify a group of metabolites that show an accumulation level that is negatively regulated with Ea titres in planta (cluster 3). In this cluster, we found mainly organic acids and fatty acids, some of which are linked metabolic precursors of defence compounds. For instance, shikimate is known to be involved in a biosynthetic pathway leading to phenylpropanoid products in plants (Dixon et al., 2002). Interestingly, a broad range of phenolic compounds have been shown to repress expression of Ea *hrp* genes in vitro (Khokhani et al., 2013). Both dehydroascorbate and glycolate are also involved in ROS accumulation processes in response to biotic stresses (O’Brien et al., 2012). β‐sitosterol is known to be involved in nonhost resistance and to play a role in membrane fluidity and nutrient efflux (Aboobucker & Suza, 2019; Wang et al., 2012). We also identified a group of metabolites that were positively associated with bacterial titres in planta (cluster 5), which included GABA. Further investigation is required to determine the link between the accumulation of these metabolites, Ea *hrp* gene expression, and defence gene activation.

Altogether, our results show that varying N supply to nonhost *Arabidopsis* affects in planta Ea virulence gene expression and that, in turn, this allows sufficient repression of the JA signalling pathway to allow an increase in bacterial titres. It would be interesting to determine whether JA signalling is affected in *Malus* under high and low N, and whether Ea virulence is affected. Although previous reports have identified culture conditions that allow in vitro induction of T3SS virulence genes, the signals that allow timely activation of these genes in planta have not been identified (Haapalainen et al., 2009). Further studies will be necessary to determine precisely how N availability affects Ea virulence gene expression in planta, in particular what signals are perceived by bacteria in planta and how they are modulated by N availability.

## 4 EXPERIMENTAL PROCEDURES

### 4.1 Plant growth

*A. thaliana* Col‐0 plants were grown for 5 weeks in sand and watered with a nutrient solution containing 2 or 10 mM ${\text{NO}}_{3}^{‐}$ (Lemaitre et al., 2008). Plants were subjected to an 8‐hr light and 16‐hr dark cycle at 21 °C (day)/18 °C (night) with 65% relative humidity.

### 4.2 Plant inoculation and measurements of bacterial titers

The bacterial strains used in this study were the wildtype strain Ea CFBP1430 (Paulin, 2000) and the *t3ss* (PMV6023) (Gaudriault et al., 1997) and *dspA/E* (M81) (Degrave et al., 2013) isogenic mutants. Syringe inoculations of leaves were performed as described previously (Zarattini et al., 2017) with a bacterial suspension of Ea with an OD_600_ of 0.1 (10^7^ cfu/ml) in water. In planta bacterial titres were determined as described previously (Degrave et al., 2008). Control leaves were mock‐inoculated with water.

### 4.3 Preinduction of Ea virulence genes

Bacteria were prepared as for standard inoculation (see above) up to the liquid culture step. Bacterial cells were then centrifuged at 3,600 × g for 10 min and washed twice in sterile water to remove the LB medium. The bacteria were resuspended in sterile water and plated on LB medium as a control or on IM solid medium, that is, M9 minimal medium (Sambrook et al., 1989) with 0.2% galactose as a carbon source (Table S1a), for 6 hr prior to plant inoculation. After that, bacteria were prepared and inoculated as described above.

### 4.4 Ea in vitro culture for N and C source utilization

Bacterial cells were grown overnight in LB medium. Bacteria were separated from LB medium by two centrifugations and resuspended in sterile water. Five hundred microlitres of bacterial suspension was added to liquid M9 minimal medium supplemented with 0.02% nicotinic acid and in which the usual nitrogen source used in M9 minimal medium, ${\text{NH}}_{4}^{+}$, had been substituted by another nitrogen source (${\text{NO}}_{3}^{‐}$ or an amino acid), at a concentration of 5 mM, as described in the text (Table S1b). To analyse C‐source utilization, galactose was substituted with the indicated C source at 5 mM (Table S1b). OD_600_ was measured at T_0_ and at different time points postincubation.

### 4.5 RNA isolation and RT‐qPCR analysis

For plant RNA extraction, five leaves of each plant (Ea‐ or mock‐treated) were collected at the indicated time point after treatment, pooled, and immediately frozen in liquid nitrogen. Total RNA was extracted from 50 mg of frozen ground leaves using TRIzol reagent (Invitrogen Life Technologies). For the RT‐qPCR analysis, first‐strand cDNA was synthesized using Superscript reverse transcriptase II (Invitrogen) from 1 µg of DNase‐treated (Invitrogen) total RNA in a 10 µl reaction volume. qPCRs were performed using SYBR Select Master Mix 2× (Applied Biosystems and Thermo Fisher Scientific) following the manufacturer's protocol. Relative expression levels were calculated following the standard curve‐based method (Larionov et al., 2005). Expression of *PP2a3* (At2g42500) (Ceccato et al., 2013) and *APT1* (At1g27450) (Vilaine et al., 2013) reference genes were used for normalization of each target gene studied. The normalized expression patterns obtained using both reference genes were similar, so only the data normalized with *PP23A* are shown. Each experiment was repeated three times independently, and within each experiment three biological replicates were analysed (i.e., three pools of five leaves for each treatment). A representative experiment is shown in Figures 4, 6, and 7. The gene‐specific primers used in this analysis are indicated in Table S2. For the Ea virulence genes expression in planta, all the steps were carried out similarly as for plant gene expression, except that the reverse transcription was performed from 5 µg total RNA using random primers (SuperScript) instead of oligo(dT). For qPCR, each Ea target gene was normalized with the *recA* (Pester et al., 2012) and *rplU* (Khokhani et al., 2013) references genes. The normalized expression patterns obtained using both reference genes were similar, so only the data normalized with *recA* are shown.

### 4.6 Detection of ROS

Hydrogen peroxide (H_2_O_2_) accumulation was detected by DAB staining. Leaves were inoculated with Ea, then sampled 2 hpi, infiltrated with a DAB solution (1 mg/ml), and placed in a Petri dish in a humid atmosphere in the dark overnight. Following discolouration with alcohol, stained leaves were observed with a Nikon Microphot‐FXA microscope at 400× magnification. Intensity of DAB staining was quantified using ImageJ software, as described previously (Launay et al., 2016). Briefly, staining intensity was measured in a square section of the inoculated half‐blade using the “Integrated density” (intdens) function of the software. The results correspond to the mean intensity of 10 leaves per condition. All experiments were performed three times.

### 4.7 Metabolite analysis

Lyophilized leaf material was used for metabolome analysis. Approximately 30 mg of the ground frozen leaf samples (mock or Ea‐inoculated leaves, harvested at 24 hpi) was analysed by an Agilent 7890A gas chromatograph coupled to an Agilent 5975C mass spectrometer as described previously (Amiour et al., 2012). Standards were injected at the beginning and end of the analysis. Data were analysed with AMDIS (http://chemdata.nist.gov/mass‐spc/amdis/) and QuanLynx software (Waters). Total free amino acids were measured from 30 mg of the ground frozen leaves by the Rosen colourimetric method with leucine as a standard (Rosen, 1957). Nitrate was determined from 20–25 mg of the ground frozen leaves as previously described (Cataldo et al., 2008). Genesis v. 1.7.6 (http://genome.tugraz.at/) was used for hierarchical cluster analysis graphical representation of the metabolic changes after log_2_ transformation. For visualization, the metabolite data were normalized by dividing each value by the median of all measurements of the experiment for a given metabolite. The subgroups were identified using the *K*‐means method.

### 4.8 SA and JA phytohormone measurement

Ea*‐* or mock‐treated rosette leaves were harvested 24 hpi and immediately frozen and ground in liquid nitrogen. For each treatment, 15 leaves from three plants were collected and 100 mg of fresh material was freeze‐dried. Hormone quantification was performed by HPLC electrospray ionization‐tandem mass spectrometry, as described previously (Le Roux et al., 2014).

## Supporting information


**FIGURE S1** Effect of nitrate supply on rosette weight. Whole rosettes of 5‐week‐old plants grown in low or high ${\text{NO}}_{3}^{‐}$ were cut and immediately weighed (*n* = 6). The observed differences in mean rosette weight were not significant according to a Mann–Whitney test (*p* < .05)

**FIGURE S2** *Erwinia amylovora* (Ea) bacterial titres in the *nahG* SA‐deficient transgenic line. Wild‐type Ea bacterial titres at 24 hr postinoculation (hpi) in the SA‐deficient *nahG* transgenic line compared to wild‐type Col‐0 plants. Plants were grown under low or high nitrate conditions for 5 weeks, then inoculated with wild‐type Ea and harvested 24 hpi. Asterisks indicate a significant difference between indicated conditions (Mann–Whitney test, *p* < .05). ns, nonsignificant

**FIGURE S3** Metabolomic analysis of *Arabidopsis* plants. Heatmaps of amino acids, sugar, and organic acids were performed by GENESIS software. The heatmap is based on the individual metabolite proportions for each biological replicate. The colour of each cell corresponds to the log_2_ ratio of each metabolite relative to the median centre. Ratio = 1, yellow; ratio < 1, blue; ratio > 1, red. Plants were grown under limiting (2 mM ${\text{NO}}_{3}^{‐}$) or full (10 mM ${\text{NO}}_{3}^{‐}$) N conditions for 5 weeks. After 5 weeks, rosette leaves were either mock‐inoculated with water (mock) or inoculated with Ea pr‐incubated for 6 hr prior to infection either in Luria‐Bertani (LB) medium or in induction medium (IM); leaves were harvested 24 hpi

**TABLE S1** Composition of bacterial culture media used in the study. (a) Composition of the induction medium (IM). (b) Composition of the liquid culture medium used to determine the N sources used by *Erwinia amylovora* (Figure 2e). (a) The N source was ammonium, nitrate or different amino acids, as specified in Figure 2e. In the M9−N control presented in Figure 2e no N source was added. (b) The C source was glucose (Figure 2e), galactose, citrate, fumarate or malate as specified in Figure 2f. In the M9−C control presented in Figure 2f no C source was added

**TABLE S2** Gene‐specific primers used in this study

## Data Availability

The data that support the findings of this study are available from the corresponding author upon reasonable request.

## References

[mpp13114-bib-0001] Aboobucker, S.I. & Suza, W.P. (2019) Why do plants convert sitosterol to stigmasterol? Frontiers in Plant Science, 10, 354. 30984220 10.3389/fpls.2019.00354 PMC6447690

[mpp13114-bib-0002] Amiour, N. , Imbaud, S. , Clément, G. , Agier, N. , Zivy, M. , Valot, B. et al. (2012) The use of metabolomics integrated with transcriptomic and proteomic studies for identifying key steps involved in the control of nitrogen metabolism in crops such as maize. Journal of Experimental Botany, 63, 5017–5033. 22936829 10.1093/jxb/ers186

[mpp13114-bib-0003] Ancona, V. , Li, W. & Zhao, Y. (2013) Alternative sigma factor RpoN and its modulation protein YhbH are indispensable for *Erwinia amylovora* virulence. Molecular Plant Pathology, 15, 58–66. 23937726 10.1111/mpp.12065 PMC6638869

[mpp13114-bib-0004] Azevedo, R.A. , Lancien, M. & Lea, P.J. (2006) The aspartic acid metabolic pathway, an exciting and essential pathway in plants. Amino Acids, 30, 143–162. 16525757 10.1007/s00726-005-0245-2

[mpp13114-bib-0005] Ballini, E. , Nguyen, T.T. & Morel, J.‐B. (2013) Diversity and genetics of nitrogen‐induced susceptibility to the blast fungus in rice and wheat. Rice, 6, 32. 24280346 10.1186/1939-8433-6-32 PMC4883689

[mpp13114-bib-0006] Bush, M. & Dixon, R. (2012) The role of bacterial enhancer binding proteins as specialized activators of σ54‐dependent transcription. Microbiology and Molecular Biology Reviews, 76, 497–529. 22933558 10.1128/MMBR.00006-12 PMC3429621

[mpp13114-bib-0007] Castro‐Moretti, F.R. , Gentzel, I.N. , Mackey, D. & Alonso, A.P. (2020) Metabolomics as an emerging tool for the study of plant–pathogen interactions. Metabolites, 10, 52–75. 10.3390/metabo10020052 PMC7074241 32013104

[mpp13114-bib-0008] Cataldo, D.A. , Maroon, M. , Schrader, L.E. & Youngs, V.L. (2008) Rapid colorimetric determination of nitrate in plant tissue by nitration of salicylic acid. Communications in Soil Science and Plant Analysis, 6, 71–80.

[mpp13114-bib-0009] Ceccato, L. , Masiero, S. , Sinha Roy, D. , Bencivenga, S. , Roig‐Villanova, I. , Ditengou, F.A. et al. (2013) Maternal control of PIN1 is required for female gametophyte development in *Arabidopsis* . PLoS One, 8, e66148. 23799075 10.1371/journal.pone.0066148 PMC3684594

[mpp13114-bib-0010] Civetta, A. , Durel, C.E. , Denancé, C. & Brisset, M.N. (2009) Two distinct major QTL for resistance to fire blight co‐localize on linkage group 12 in apple genotypes “Evereste” and *Malus* floribunda clone 821. Genome, 52, 139–147. 19234562 10.1139/g08-111

[mpp13114-bib-0011] Daugaard, H. , Sørensen, L. & Løschenkohl, B. (2016) Effect of plant spacing, nitrogen fertilisation, post‐harvest defoliation and finger harrowing in the control of *Botrytis cinerea* Pers. in strawberry. European Journal of Horticultural Science, 68, 77–82.

[mpp13114-bib-0012] Dechorgnat, J. , Patrit, O. , Krapp, A. , Fagard, M. & Daniel‐Vedele, F. (2012) The *AtNRT2.6* gene is involved in the response of *Arabidopsis thaliana* to *Erwinia amylovora* . PLoS One, 7, e42491. 22880003 10.1371/journal.pone.0042491 PMC3413667

[mpp13114-bib-0013] Degrave, A. , Fagard, M. , Perino, C. , Brisset, M.‐N. , Gaubert, S. , Laroche, S. et al. (2008) *Erwinia amylovora* type III‐secreted proteins trigger cell death and defense responses in *Arabidopsis thaliana* . Molecular Plant‐Microbe Interactions, 21, 1076–1086. 18616404 10.1094/MPMI-21-8-1076

[mpp13114-bib-0014] Degrave, A. , Moreau, M. , Launay, A. , Barny, M.‐A. , Brisset, M.‐N. , Patrit, O. et al. (2013) The bacterial effector DspA/E is toxic in *Arabidopsis thaliana* and is required for multiplication and survival of fire blight pathogen. Molecular Plant Pathology, 14, 506–517. 23634775 10.1111/mpp.12022 PMC6638835

[mpp13114-bib-0015] Dietrich, C.R. , Ploß, K. & Heil, K. (2004) Constitutive and induced resistance to pathogens in *Arabidopsis thaliana* depends on nitrogen supply. Plant, Cell and Environment, 27, 896–906.

[mpp13114-bib-0016] Dixon, R.A. , Achnine, L. , Kota, P. , Liu, C.‐J. , Reddy, M.S.S. & Wang, L. (2002) The phenylpropanoid pathway and plant defence – a genomics perspective. Molecular Plant Pathology, 3, 371–390. 20569344 10.1046/j.1364-3703.2002.00131.x

[mpp13114-bib-0017] Dordas, C. (2008) Role of nutrients in controlling plant diseases in sustainable agriculture. A review. Agronomy for Sustainable Development, 28, 33–46.

[mpp13114-bib-0018] Dugé de Bernonville, T.D. , Gaucher, M. , Flors, V. , Gaillard, S. , Paulin, J.‐P. , Dat, J.F. et al. (2012) T3SS‐dependent differential modulations of the jasmonic acid pathway in susceptible and resistant genotypes of *Malus* spp. challenged with *Erwinia amylovora* . Plant Science, 188–189, 1–9. 10.1016/j.plantsci.2012.02.009 22525238

[mpp13114-bib-0019] Fagard, M. , Launay, A. , Clement, G. , Courtial, J. , Dellagi, A. , Farjad, M. et al. (2014) Nitrogen metabolism meets phytopathology. Journal of Experimental Botany, 65, 5643–5656. 25080088 10.1093/jxb/eru323

[mpp13114-bib-0020] Fallahi, E. & Mohan, S.K. (2000) Influence of nitrogen and rootstock on tree growth, precocity, fruit quality, leaf mineral nutrients, and fire blight in ‘Scarlet Gala’ apple. Hortechnology, 10, 589–592.

[mpp13114-bib-0021] Galán, J.E. & Wolf‐Watz, H. (2006) Protein delivery into eukaryotic cells by type III secretion machines. Nature, 444, 567–573. 17136086 10.1038/nature05272

[mpp13114-bib-0022] Gaudriault, S. , Malandrin, L. , Paulin, J.P. & Barny, M.A. (1997) DspA, an essential pathogenicity factor of *Erwinia amylovora* showing homology with AvrE of *Pseudomonas syringae*, is secreted via the Hrp secretion pathway in a DspB‐dependent way. Molecular Microbiology, 26, 1057–1069. 9426142 10.1046/j.1365-2958.1997.6442015.x

[mpp13114-bib-0023] Gupta, K.J. , Brotman, Y. , Segu, S. , Zeier, T. , Zeier, J. , Persijn, S.T. et al. (2012) The form of nitrogen nutrition affects resistance against *Pseudomonas syringae* pv. *phaseolicola* in tobacco. Journal of Experimental Botany, 64, 553–568. 23230025 10.1093/jxb/ers348 PMC3542047

[mpp13114-bib-0024] Haapalainen, M. , van Gestel, K. , Pirhonen, M. & Taira, S. (2009) Soluble plant cell signals induce the expression of the Type III secretion system of *Pseudomonas syringae* and upregulate the production of pilus protein HrpA. Molecular Plant‐Microbe Interactions, 22, 282–290. 19245322 10.1094/MPMI-22-3-0282

[mpp13114-bib-0025] He, S.Y. , Nomura, K. & Whittam, T.S. (2004) Type III protein secretion mechanism in mammalian and plant pathogens. Biochimica et Biophysica Acta, 1694, 181–206. 15546666 10.1016/j.bbamcr.2004.03.011

[mpp13114-bib-0026] Hildebrandt, T.M. , Nesi, A.N. , Araújo, W.L. & Braun, H.‐P. (2015) Amino acid catabolism in plants. Molecular Plant, 8, 1563–1579. 26384576 10.1016/j.molp.2015.09.005

[mpp13114-bib-0027] Hoffland, E. , Jeger, M.J. & van Beusichem, M.L. (2000) Effect of nitrogen supply rate on disease resistance in tomato depends on the pathogen. Plant and Soil, 218, 239–247.

[mpp13114-bib-0028] Holtappels, M. , Noben, J.‐P. & Valcke, R. (2016) Virulence of *Erwinia amylovora*, a prevalent apple pathogen: Outer membrane proteins and type III secreted effectors increase fitness and compromise plant defenses. Proteomics, 16, 2377–2390. 27345300 10.1002/pmic.201500513

[mpp13114-bib-0029] Khokhani, D. , Zhang, C. , Li, Y. , Wang, Q.i. , Zeng, Q. , Yamazaki, A. et al. (2013) Discovery of plant phenolic compounds that act as type III secretion system inhibitors or inducers of the fire blight pathogen, *Erwinia amylovora* . Applied and Environmental Microbiology, 79, 5424–5436. 23770912 10.1128/AEM.00845-13 PMC3754148

[mpp13114-bib-0030] Krapp, A. , Berthomé, R. , Orsel, M. , Mercey‐Boutet, S. , Yu, A. , Castaings, L. et al. (2011) *Arabidopsis* roots and shoots show distinct temporal adaptation patterns toward nitrogen starvation. Plant Physiology, 157, 1255–1282. 21900481 10.1104/pp.111.179838 PMC3252138

[mpp13114-bib-0031] Larionov, A. , Krause, A. & Miller, W. (2005) A standard curve based method for relative real time PCR data processing. BMC Bioinformatics, 6, 62. 15780134 10.1186/1471-2105-6-62 PMC1274258

[mpp13114-bib-0032] Launay, A. , Patrit, O. , Wénès, E. & Fagard, M. (2016) DspA/E contributes to apoplastic accumulation of ROS in non‐host *A. thaliana* . Frontiers in Plant Science, 7, 545. 27200021 10.3389/fpls.2016.00545 PMC4845087

[mpp13114-bib-0033] Le Roux, C. , Del Prete, S. , Boutet‐Mercey, S. , Perreau, F. , Balagué, C. , Roby, D. et al. (2014) The hnRNP‐Q protein LIF2 participates in the plant immune response. PLoS One, 9, e99343. 24914891 10.1371/journal.pone.0099343 PMC4051675

[mpp13114-bib-0034] Lecompte, F. , Abro, M.A. & Nicot, P.C. (2010) Contrasted responses of *Botrytis cinerea* isolates developing on tomato plants grown under different nitrogen nutrition regimes. Plant Pathology, 59, 891–899.

[mpp13114-bib-0035] Lemaitre, T. , Gaufichon, L. , Boutet‐Mercey, S. , Christ, A. & Masclaux‐Daubresse, C. (2008) Enzymatic and metabolic diagnostic of nitrogen deficiency in *Arabidopsis thaliana* Wassileskija accession. Plant and Cell Physiology, 49, 1056–1065. 18508804 10.1093/pcp/pcn081

[mpp13114-bib-0036] Malnoy, M. , Martens, S. , Norelli, J.L. , Barny, M.‐A. , Sundin, G.W. , Smits, T.H.M. et al. (2012) Fire blight: applied genomic insights of the pathogen and host. Annual Review of Phytopathology, 50, 475–494. 10.1146/annurev-phyto-081211-172931 22702352

[mpp13114-bib-0037] McCraw, S.L. , Park, D.H. , Jones, R. , Bentley, M.A. , Rico, A. , Ratcliffe, R.G. et al. (2016) GABA (γ‐aminobutyric acid) uptake via the GABA permease GabP represses virulence gene expression in *Pseudomonas syringae* pv. *tomato* DC3000. Molecular Plant‐Microbe Interactions, 29, 938–949. 28001093 10.1094/MPMI-08-16-0172-R

[mpp13114-bib-0038] Moreau, M. , Degrave, A. , Vedel, R. , Bitton, F. , Patrit, O. , Renou, J.‐P. et al. (2012) EDS1 contributes to nonhost resistance of *Arabidopsis thaliana* against *Erwinia amylovora* . Molecular Plant‐Microbe Interactions, 25, 421–430. 22316300 10.1094/MPMI-05-11-0111

[mpp13114-bib-0039] O’Brien, J.A. , Daudi, A. , Butt, V.S. & Paul Bolwell, G. (2012) Reactive oxygen species and their role in plant defence and cell wall metabolism. Planta, 236, 765–779. 22767200 10.1007/s00425-012-1696-9

[mpp13114-bib-0040] Parravicini, G. , Gessler, C. , Denancé, C. , Lassere‐Zuber, P. , Vergne, E. , Brisset, M.‐N. et al. (2011) Identification of serine/threonine kinase and nucleotide‐binding site‐leucine‐rich repeat (NBS‐LRR) genes in the fire blight resistance quantitative trait locus of apple cultivar “Evereste”. Molecular Plant Pathology, 12, 493–505. 21535354 10.1111/j.1364-3703.2010.00690.x PMC6640535

[mpp13114-bib-0041] Paulin, J.‐P. (2000) *Erwinia amylovora*: general characteristics, biochemistry and serology. In: Vanneste, J.L. (Ed.) Fire blight: the disease and its causative agent, Erwinia amylovora. Wallingford, UK: CABI, pp. 87–115.

[mpp13114-bib-0042] Pester, D. , Milčevičová, R. , Schaffer, J. , Wilhelm, E. & Blümel, S. (2012) *Erwinia amylovora* expresses fast and simultaneously *hrp/dsp* virulence genes during flower infection on apple trees. PLoS One, 7, e32583. 22412891 10.1371/journal.pone.0032583 PMC3295760

[mpp13114-bib-0043] Rosen, H. (1957) A modified ninhydrin colorimetric analysis for amino acids. Archives of Biochemistry and Biophysics, 67, 10–15. 13412116 10.1016/0003-9861(57)90241-2

[mpp13114-bib-0044] Royer, M. , Larbat, R. , Le Bot, J. , Adamowicz, S. & Robin, C. (2013) Is the C: N ratio a reliable indicator of C allocation to primary and defence‐related metabolisms in tomato? Phytochemistry, 88, 25–33. 23312460 10.1016/j.phytochem.2012.12.003

[mpp13114-bib-0045] Sambrook, J. , Frisch, E. & Maniatis, T. (1989) Molecular cloning. A laboratory manual, 2nd edition. Cold Spring Harbor, NY: Cold Spring Harbor Laboratory Press.

[mpp13114-bib-0046] Seifi, H.S. , Van Bockhaven, J. , Angenon, G. & Höfte, M. (2013) Glutamate metabolism in plant disease and defense: friend or foe? Molecular Plant‐Microbe Interactions, 26, 475–485. 23342972 10.1094/MPMI-07-12-0176-CR

[mpp13114-bib-0047] Snoeijers, S. , Pérez‐García, A. , Joosten, M.H.A.J. & de Wit, P. (2000) The effect of nitrogen on disease development and gene expression in bacterial and fungal plant pathogens. European Journal of Plant Pathology, 106, 493–506.

[mpp13114-bib-0048] Soulié, M.‐C. , Mia Koka, S. , Floch, K. , Vancostenoble, B. , Barbé, D. , Daviere, A. et al. (2020) Plant nitrogen supply affects the *Botrytis cinerea* infection process and modulates known and novel virulence factors. Molecular Plant Pathology, 21, 1436–1450. 32939948 10.1111/mpp.12984 PMC7549004

[mpp13114-bib-0049] Vilaine, F. , Kerchev, P. , Clément, G. , Batailler, B. , Cayla, T. , Bill, L. et al. (2013) Increased expression of a phloem membrane protein encoded by NHL26 alters phloem export and sugar partitioning in *Arabidopsis* . The Plant Cell, 25, 1689–1708. 23715470 10.1105/tpc.113.111849 PMC3694700

[mpp13114-bib-0050] Vogt, I. , Wöhner, T. , Richter, K. , Flachowsky, H. , Sundin, G.W. , Wensing, A. et al. (2013) Gene‐for‐gene relationship in the host–pathogen system *Malus* × *robusta*–*Erwinia amylovora* . New Phytologist, 197, 1262–1275. 10.1111/nph.12094 23301854

[mpp13114-bib-0051] Wang, K. , Senthil‐Kumar, M. , Ryu, C.‐M. , Kang, L. & Mysore, K.S. (2012) Phytosterols play a key role in plant innate immunity against bacterial pathogens by regulating nutrient efflux into the apoplast. Plant Physiology, 158, 1789–1802. 22298683 10.1104/pp.111.189217 PMC3320186

[mpp13114-bib-0052] Ward, J.L. , Forcat, S. , Beckmann, M. , Bennett, M. , Miller, S.J. , Baker, J.M. et al. (2010) The metabolic transition during disease following infection of *Arabidopsis thaliana* by *Pseudomonas syringae* pv. *tomato* . The Plant Journal, 63, 443–457. 20497374 10.1111/j.1365-313X.2010.04254.x

[mpp13114-bib-0053] Wei, Z.M. , Sneath, B.J. & Beer, S.V. (1992) Expression of *Erwinia amylovora hrp* genes in response to environmental stimuli. Journal of Bacteriology, 174, 1875–1882. 1372313 10.1128/jb.174.6.1875-1882.1992 PMC205791

[mpp13114-bib-0054] Wöhner, T. , Richter, K. , Sundin, G.W. , Zhao, Y. , Stockwell, V.O. , Sellmann, J. et al. (2018) Inoculation of *Malus* genotypes with a set of *Erwinia amylovora* strains indicates a gene‐for‐gene relationship between the effector gene *eop1* and both *Malus floribunda* 821 and *Malus* ‘Evereste’. Plant Pathology, 67, 938–947.

[mpp13114-bib-0055] Yu, X. , Lund, S.P. , Scott, R.A. , Greenwald, J.W. , Records, A.H. , Nettleton, D. et al. (2013) Transcriptional responses of *Pseudomonas syringae* to growth in epiphytic versus apoplastic leaf sites. Proceedings of the National Academy of Sciences of the United States of America, 110, E425–E434. 23319638 10.1073/pnas.1221892110 PMC3562829

[mpp13114-bib-0056] Zarattini, M. , Farjad, M. , Launay, A. , Cannella, D. , Soulié, M.‐C. , Bernacchia, G. et al. (2021) Every cloud has a silver lining: how abiotic stresses affect gene expression in plant–pathogen interactions. Journal of Experimental Botany, 72, 1020–1033. 33188434 10.1093/jxb/eraa531 PMC7904152

[mpp13114-bib-0057] Zarattini, M. , Launay, A. , Farjad, M. , Wénès, E. , Taconnat, L. , Boutet, S. et al. (2017) The bile acid deoxycholate elicits defences in *Arabidopsis* and reduces bacterial infection. Molecular Plant Pathology, 18, 540–554. 27085087 10.1111/mpp.12416 PMC6638291

