# Supplementary figures and images for: Plant nitrate supply regulates Erwinia amylovora virulence gene expression in Arabidopsis
Source: Mol Plant Pathol. 2021 Aug 12;22(11):1332–46. doi: 10.1111/mpp.13114 (PMC8518577; doi:10.1111/mpp.13114)

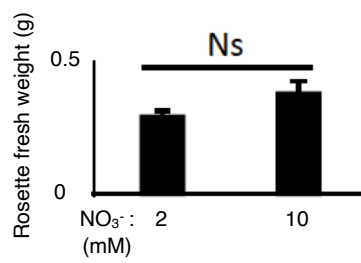

Figure S1

Supplement: Supplementary file 1 — FIGURE S1 Effect of nitrate supply on rosette weight. Whole rosettes of 5‐week‐old plants grown in low or high NO3‐ were cut and immediately weighed (n = 6). The observed differences in mean rosette weight were not significant according to a Mann–Whitney test (p < .05) [file MPP-22-1332-s001.pdf]

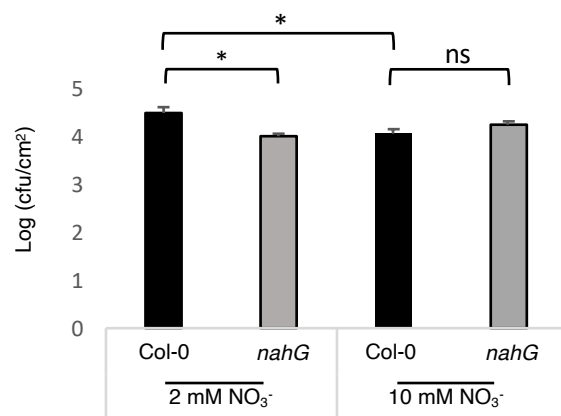

Figure S2

Supplement: Supplementary file 2 — FIGURE S2 Erwinia amylovora (Ea) bacterial titres in the nahG SA‐deficient transgenic line. Wild‐type Ea bacterial titres at 24 hr postinoculation (hpi) in the SA‐deficient nahG transgenic line compared to wild‐type Col‐0 plants. Plants were grown under low or high nitrate conditions for 5 weeks, then inoculated with wild‐type Ea and harvested 24 hpi. Asterisks indicate a significant difference between indicated conditions (Mann–Whitney test, p < .05). ns, nonsignificant [file MPP-22-1332-s004.pdf]

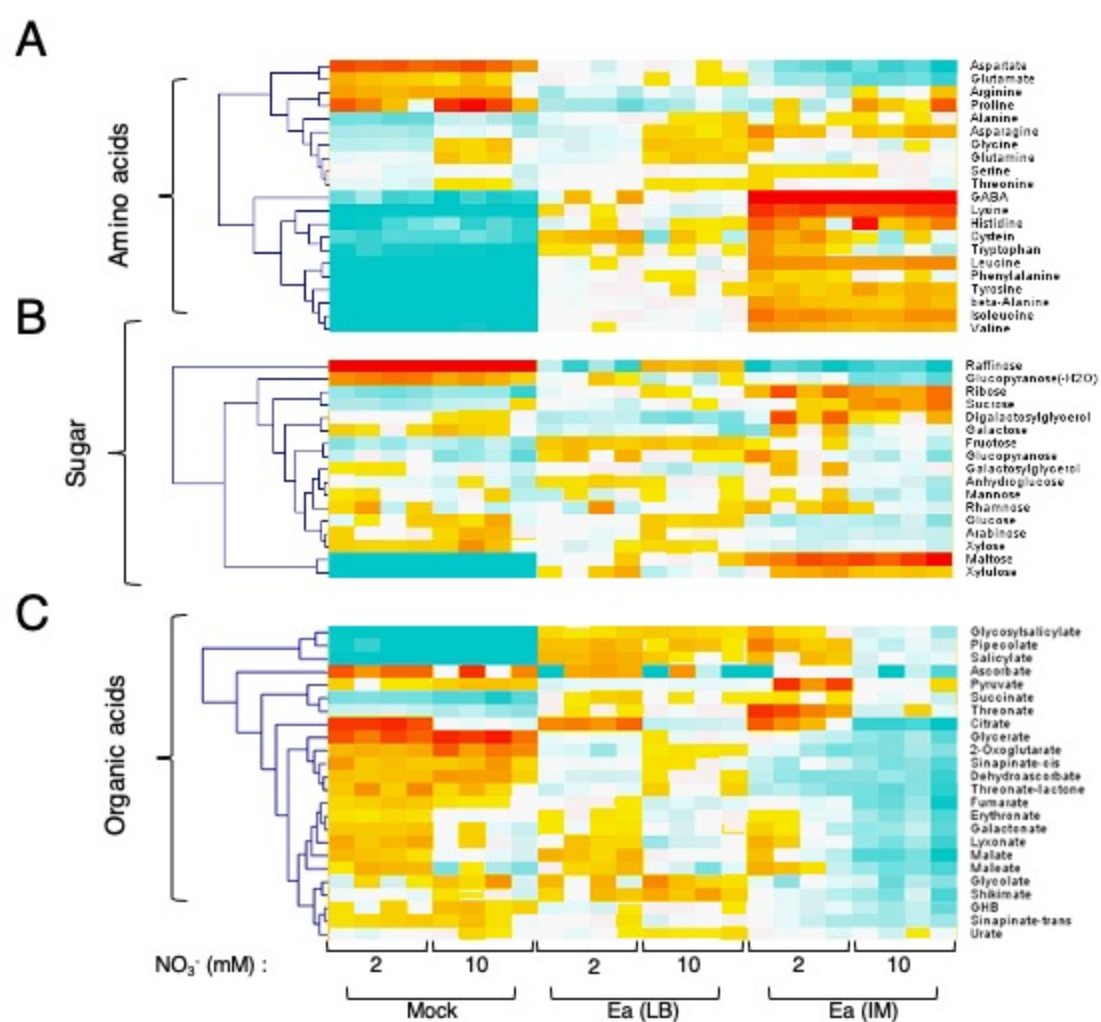

Figure S3

Supplement: Supplementary file 3 — FIGURE S3 Metabolomic analysis of Arabidopsis plants. Heatmaps of amino acids, sugar, and organic acids were performed by GENESIS software. The heatmap is based on the individual metabolite proportions for each biological replicate. The colour of each cell corresponds to the log2 ratio of each metabolite relative to the median centre. Ratio = 1, yellow; ratio < 1, blue; ratio > 1, red. Plants were grown under limiting (2 mM NO3‐) or full (10 mM NO3‐) N conditions for 5 weeks. After 5 weeks, rosette leaves were either mock‐inoculated with water (mock) or inoculated with Ea pr‐incubated for 6 hr prior to infection either in Luria‐Bertani (LB) medium or in induction medium (IM); leaves were harvested 24 hpi [file MPP-22-1332-s005.pdf]

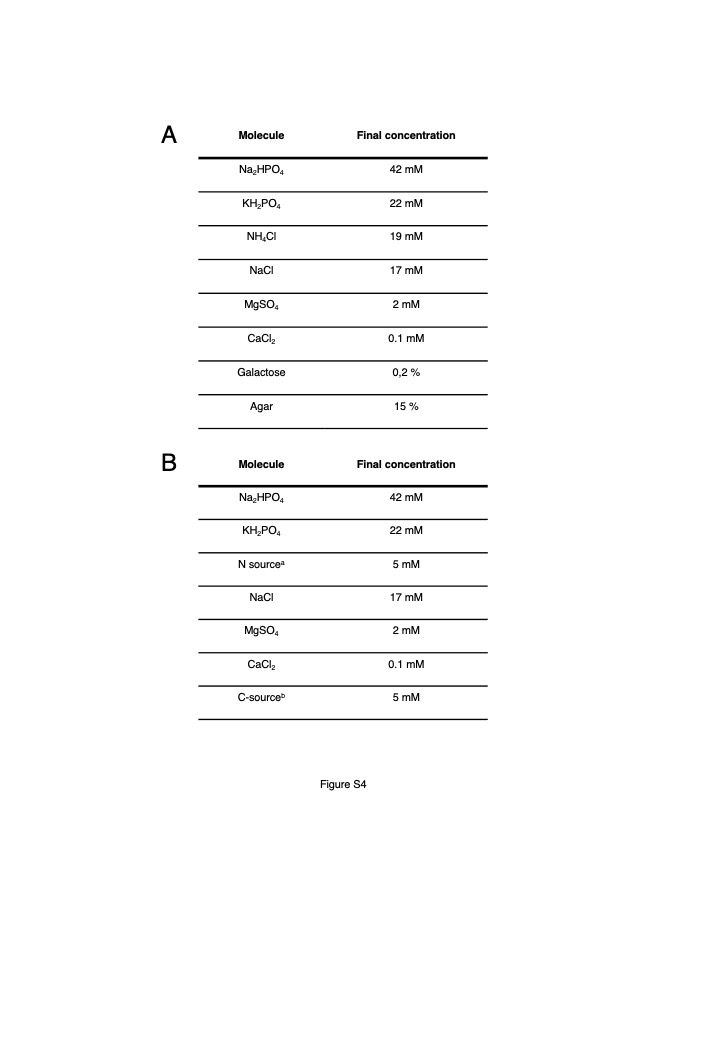

Supplement: Supplementary file 4 — TABLE S1 Composition of bacterial culture media used in the study. (a) Composition of the induction medium (IM). (b) Composition of the liquid culture medium used to determine the N sources used by Erwinia amylovora (Figure 2e). (a) The N source was ammonium, nitrate or different amino acids, as specified in Figure 2e. In the M9−N control presented in Figure 2e no N source was added. (b) The C source was glucose (Figure 2e), galactose, citrate, fumarate or malate as specified in Figure 2f. In the M9−C control presented in Figure 2f no C source was added [file MPP-22-1332-s003.docx]
