# Supplementary material for: Plant nitrate supply regulates Erwinia amylovora virulence gene expression in Arabidopsis
Source: Mol Plant Pathol. 2021 Aug 12;22(11):1332–46. doi: 10.1111/mpp.13114 (PMC8518577; doi:10.1111/mpp.13114)
Supplement: Supplementary file 5 — TABLE S2 Gene‐specific primers used in this study [file MPP-22-1332-s002.pdf]

A. thaliana genes	LP	RP
AOC	GATTCGTTCCTTGCCATCAC	AACCTGTCCGTAGGCACCTT
AOS	GACCAATCAAAGACCGTTGG	CGGATTCGTGATTTGAAGAAC
COI1	CTGACTTCCCTTAGGTACTTGTGG	CAACTCGTCTCGGAGGAATCAAC
EDS1	AGATCAATGGCGTTTGAAGCTCTTA	ACCCCATCATGAGACCATTTCAATC
ICS1	CAGTCCGAAAGACGACCTCGAGTT	GCTGGAGTTGGATGCAGAGCAGCC
JAR1	CGTTCCCTTCTCCACAATCC	GGGTTCTTGGTGAATGTTGC
NPR1	AGGGGATATACGGTGCTTCA	ATGCACTTGCACCTTTTTCC
PAD4	CATGGACGATTGTCGATTCG	ATTTGCGGTGTTGCATGAAC
PDF1.2	TGATCCATGTTTGGCTCCTTCA	AAGCCAAGTGGGACATGGTCA
PR1	TCTTCCCTCGAAAGCTCAAGA	GTGCCTGGTTGTGAACCCTTA

E.amylovora genes	LP	RP
amsG	GCTTTATGGCACGGATATGG	CCAACGAGATCGAAGGTACG
dspA/E	TCCAGCGAGGGCATAATACT	ACAACCGTACCCTGCAAAAC
hrpA	TACAAGCGCAAGCACTTCAG	GAGTCCATTTTGCCATCCAG
hrpL	TTAAGGCAATGCCAAACACC	GACGCGTGCATCATTTTATT
hrpN	GCTTTTGCCCATGATTTGTC	CAACCCGTTCTTTCGTCAAT
hrpS	AATGCTACGCGTGCTGGAAA	AACAATGGCGTTTGCGTTGC
rpoN	AAGCGGTACTGAAACGGGTA	GCATCAGACTGCGAAAATCA

Table S2
